# Trehalose dihydrate from *Tremella fuciformis*

**DOI:** 10.1107/S1600536812031947

**Published:** 2012-07-21

**Authors:** Wei Liu, Jun Yan, Qin Song, Xiao-Jun Gou, Feng-Zheng Chen

**Affiliations:** a The Key Laboratory of Medicinal and Edible Plants Resources Development of, Sichuan Education Commission, Chengdu University, Chengdu 610106, People’s Republic of China

## Abstract

The title compound, C_12_H_22_O_11_·2H_2_O {systematic name: 6,6′-oxybis[2-(hy­droxy­meth­yl)-3,4,5,6-tetra­hydro-2*H*-pyran-3,4,5-triol] dihydrate}, is a disaccharide, which was isolated from *Tremella fuciformis*. The mol­ecule contains two six-membered rings, both of which adopt a chair conformation. Extensive O—H⋯O hydrogen bonds occur in the crystal structure.

## Related literature

For the structure of the title compound established from the NMR and MS data, see: Qing & Liu (2012 ▶).
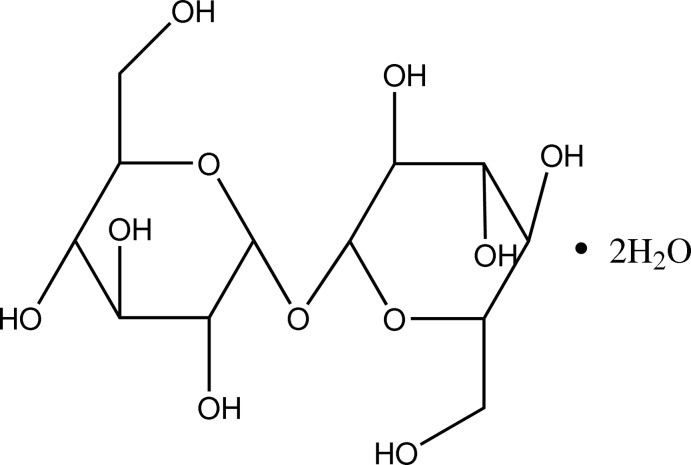


## Experimental

### 

#### Crystal data

C_12_H_22_O_11_·2H_2_O
*M* *_r_* = 378.33
Orthorhombic, 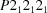

*a* = 7.6012 (3) Å
*b* = 12.2380 (4) Å
*c* = 17.8839 (6) Å
*V* = 1663.64 (9) Å^3^
*Z* = 4
Mo *K*α radiation
μ = 0.14 mm^−1^
*T* = 293 K
0.40 × 0.40 × 0.35 mm

#### Data collection

Oxford Diffraction Xcalibur Eos diffractometer
4079 measured reflections
1698 independent reflections
1492 reflections with *I* > 2σ(*I*)
*R*_int_ = 0.025

#### Refinement

*R*[*F* ^2^ > 2σ(*F* ^2^)] = 0.034
*wR*(*F* ^2^) = 0.074
*S* = 1.05
1698 reflections
274 parameters
H atoms treated by a mixture of independent and constrained refinement
Δρ_max_ = 0.16 e Å^−3^
Δρ_min_ = −0.19 e Å^−3^

### 

Data collection: *CrysAlis PRO* (Oxford Diffraction, 2009 ▶); cell refinement: *CrysAlis PRO*; data reduction: *CrysAlis PRO*; program(s) used to solve structure: *SHELXTL* (Sheldrick, 2008 ▶); program(s) used to refine structure: *SHELXTL*; molecular graphics: *SHELXTL*; software used to prepare material for publication: *SHELXTL*.

## Supplementary Material

Crystal structure: contains datablock(s) I, global. DOI: 10.1107/S1600536812031947/xu5574sup1.cif

Structure factors: contains datablock(s) I. DOI: 10.1107/S1600536812031947/xu5574Isup2.hkl

Supplementary material file. DOI: 10.1107/S1600536812031947/xu5574Isup3.cml

Additional supplementary materials:  crystallographic information; 3D view; checkCIF report

## Figures and Tables

**Table 1:** Hydrogen-bond geometry (Å, °)

*D*—H⋯*A*	*D*—H	H⋯*A*	*D*⋯*A*	*D*—H⋯*A*
O2—H2⋯O13	0.83 (5)	1.91 (5)	2.727 (3)	168 (4)
O3—H3⋯O9^i^	0.76 (4)	2.05 (4)	2.754 (3)	153 (4)
O4—H4⋯O1^ii^	0.77 (4)	2.21 (4)	2.889 (3)	147 (3)
O5—H5⋯O10^iii^	0.87 (4)	1.88 (4)	2.726 (3)	163 (3)
O8—H8⋯O12^iv^	0.82 (4)	1.98 (4)	2.765 (3)	161 (4)
O9—H9⋯O4^i^	0.75 (4)	2.16 (4)	2.907 (3)	174 (4)
O10—H10⋯O12	0.75 (5)	2.14 (4)	2.887 (3)	170 (5)
O11—H11⋯O5^v^	0.78 (4)	1.94 (4)	2.709 (3)	173 (4)
O12—H12*C*⋯O2	0.89 (4)	1.87 (4)	2.766 (3)	175 (3)
O12—H12*D*⋯O8^vi^	0.72 (5)	1.99 (5)	2.709 (4)	171 (4)
O13—H13*A*⋯O3^vii^	0.78 (5)	2.04 (5)	2.801 (4)	169 (6)
O13—H13*B*⋯O11	0.88 (5)	1.89 (5)	2.765 (3)	179 (6)

Symmetry codes: (i) 
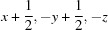
; (ii) 
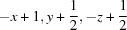
; (iii) 
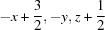
; (iv) 
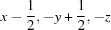
; (v) 
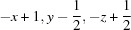
; (vi) 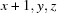; (vii) 
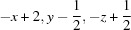
.

**Fig. 1.**
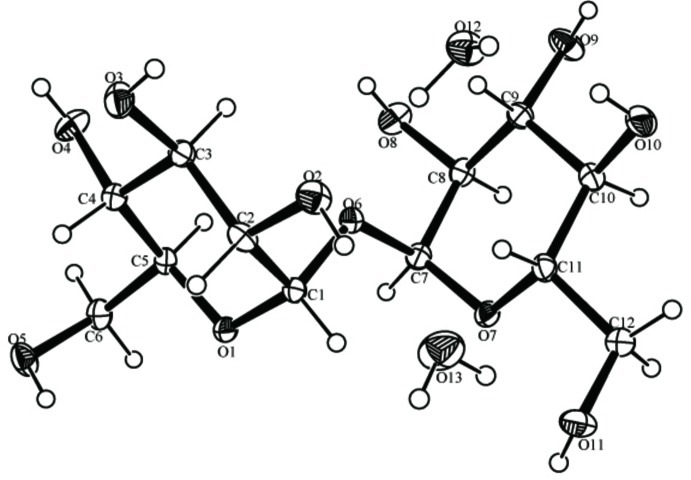
The molecular structure of the title compound with 30% probability displacement ellipsoids.

**Fig. 2.**
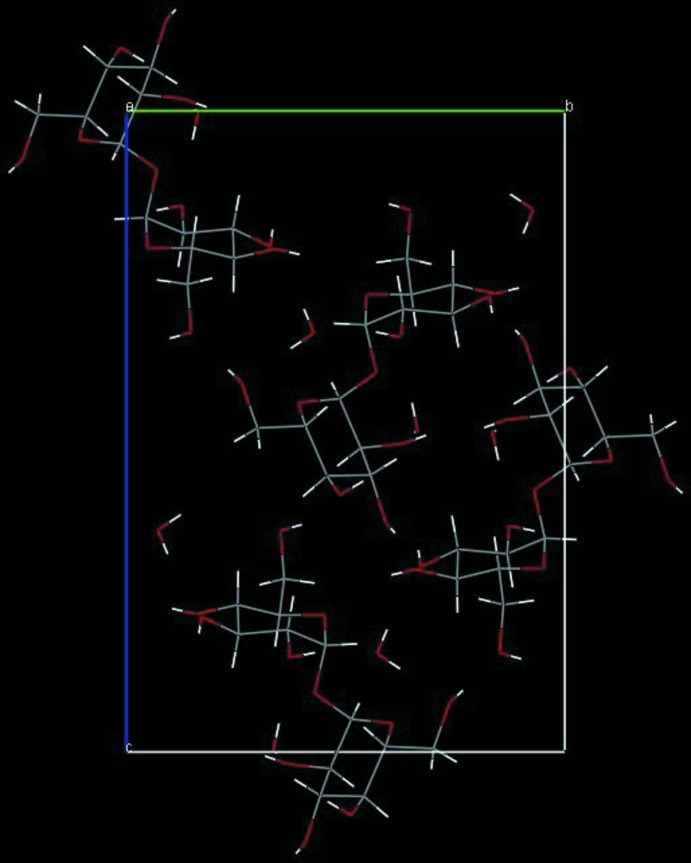
Molecular packing of the title compound.

## supplementary crystallographic information

### Comment

The title compound, trehalose, was previously isolated from *Lepista multiformis*, and its structure was established from the NMR and MS data (Qing & Liu, 2012). In our recent investigation, it was isolation from the *Tremella fuciformis* collected in the Tongjiang county, Sichuan Province of China in September, 2011 for the first time, and its crystal structure was determined.

The molecular structure of the title compound is shown in Fig. 1. The boat conformations six-membered ringsA (C1/C2/C3/C4/C5/O) and B (C7/C8/C9/C10/C11/O) link with the mid-poit of the C—O—C bond.

The lattice water molecule link with the organic molecule *via* classic O—H···O hydrogen is present in the crystal structure (Table 1).

The intermolecular hydrogen bonds may be effective in the stabilization of the structure.

### Experimental

Air-dried and powdered of *Tremella fuciformis* (5 kg) was extract with ethanol. The crude extract obtained after evaporation of the solvent was subjected to conventional purification procedures and resulting in the isolation of trehalose. The crystals suitable for X-ray structure analysis was obtained by slow evaporation from the solution of hydrous ethanol at room temperature.

### Refinement

Hydroxyl H atoms were located in a difference Fourier map and refined isotropically. Other H atoms were located geometrically with C—H = 0.97–0.98 Å, and refined using a riding model with U_iso_(H) = 1.2U_eq_(C). The absolute configuration has not been determined as no significant anomalous scatterings.

### Crystal data

**Table:**

C_12_H_22_O_11_·2H_2_O	*D*_x_ = 1.510 Mg m^−^^3^
*M**_r_* = 378.33	Mo *K*α radiation, λ = 0.71073 Å
Orthorhombic, *P*2_1_2_1_2_1_	Cell parameters from 1713 reflections
*a* = 7.6012 (3) Å	θ = 2.9–28.7°
*b* = 12.2380 (4) Å	µ = 0.14 mm^−^^1^
*c* = 17.8839 (6) Å	*T* = 293 K
*V* = 1663.64 (9) Å^3^	Block, colorless
*Z* = 4	0.40 × 0.40 × 0.35 mm
*F*(000) = 808	

### Data collection

**Table:**

Oxford Diffraction Xcalibur Eos diffractometer	1492 reflections with *I* > 2σ(*I*)
Radiation source: fine-focus sealed tube	*R*_int_ = 0.025
Graphite monochromator	θ_max_ = 25.0°, θ_min_ = 2.9°
Detector resolution: 16.08 pixels mm^-1^	*h* = −4→9
ω scans	*k* = −7→14
4079 measured reflections	*l* = −21→19
1698 independent reflections	

### Refinement

**Table:**

Refinement on *F*^2^	Primary atom site location: structure-invariant direct methods
Least-squares matrix: full	Secondary atom site location: difference Fourier map
*R*[*F*^2^ > 2σ(*F*^2^)] = 0.034	Hydrogen site location: inferred from neighbouring sites
*wR*(*F*^2^) = 0.074	H atoms treated by a mixture of independent and constrained refinement
*S* = 1.05	*w* = 1/[σ^2^(*F*_o_^2^) + (0.0383*P*)^2^] where *P* = (*F*_o_^2^ + 2*F*_c_^2^)/3
1698 reflections	(Δ/σ)_max_ = 0.002
274 parameters	Δρ_max_ = 0.16 e Å^−^^3^
0 restraints	Δρ_min_ = −0.19 e Å^−^^3^

### Special details

**Table:**

Geometry. All esds (except the esd in the dihedral angle between two l.s. planes) are estimated using the full covariance matrix. The cell esds are taken into account individually in the estimation of esds in distances, angles and torsion angles; correlations between esds in cell parameters are only used when they are defined by crystal symmetry. An approximate (isotropic) treatment of cell esds is used for estimating esds involving l.s. planes.
Refinement. Refinement of F^2^ against ALL reflections. The weighted R-factor wR and goodness of fit S are based on F^2^, conventional R-factors R are based on F, with F set to zero for negative F^2^. The threshold expression of F^2^ > 2sigma(F^2^) is used only for calculating R-factors(gt) etc. and is not relevant to the choice of reflections for refinement. R-factors based on F^2^ are statistically about twice as large as those based on F, and R- factors based on ALL data will be even larger.

### Fractional atomic coordinates and isotropic or equivalent isotropic displacement parameters (Å^2^)

**Table:**

	*x*	*y*	*z*	*U*_iso_*/*U*_eq_	
O1	0.4981 (3)	0.04792 (14)	0.21413 (9)	0.0228 (4)	
O2	0.9359 (3)	0.12683 (19)	0.14721 (11)	0.0313 (5)	
O3	0.8041 (3)	0.32880 (18)	0.21134 (12)	0.0350 (6)	
O4	0.4253 (3)	0.34117 (18)	0.21507 (13)	0.0350 (5)	
O5	0.3163 (3)	0.14840 (19)	0.34554 (10)	0.0332 (5)	
O6	0.6033 (3)	0.07053 (14)	0.09141 (9)	0.0225 (4)	
O7	0.6186 (3)	−0.10804 (14)	0.04493 (10)	0.0237 (5)	
O8	0.3693 (3)	0.13076 (18)	−0.01895 (12)	0.0323 (5)	
O9	0.6012 (3)	0.0916 (2)	−0.14203 (12)	0.0371 (6)	
O10	0.9303 (3)	−0.01309 (19)	−0.09912 (13)	0.0337 (5)	
O11	0.9290 (3)	−0.23738 (18)	0.07721 (11)	0.0326 (5)	
O12	1.0200 (4)	0.1657 (2)	−0.00091 (14)	0.0356 (6)	
O13	1.0982 (4)	−0.0717 (2)	0.15388 (17)	0.0503 (7)	
C1	0.6474 (4)	0.0457 (2)	0.16644 (14)	0.0227 (6)	
H1	0.7024	−0.0267	0.1687	0.027*	
C2	0.7790 (4)	0.1315 (2)	0.19079 (14)	0.0242 (7)	
H2A	0.8093	0.1189	0.2433	0.029*	
C3	0.6952 (4)	0.2434 (2)	0.18370 (14)	0.0235 (6)	
H3A	0.6670	0.2575	0.1311	0.028*	
C4	0.5269 (4)	0.2458 (2)	0.22963 (14)	0.0232 (6)	
H4A	0.5581	0.2447	0.2828	0.028*	
C5	0.4050 (4)	0.1502 (2)	0.21362 (14)	0.0211 (6)	
H5A	0.3528	0.1606	0.1640	0.025*	
C6	0.2581 (4)	0.1401 (3)	0.27022 (14)	0.0273 (7)	
H6A	0.1720	0.1970	0.2608	0.033*	
H6B	0.2002	0.0702	0.2634	0.033*	
C7	0.5152 (4)	−0.0136 (2)	0.05112 (14)	0.0230 (6)	
H7	0.4050	−0.0317	0.0767	0.028*	
C8	0.4733 (4)	0.0350 (2)	−0.02570 (15)	0.0243 (6)	
H8A	0.4056	−0.0192	−0.0540	0.029*	
C9	0.6428 (4)	0.0571 (2)	−0.06789 (13)	0.0231 (6)	
H9A	0.7053	0.1166	−0.0427	0.028*	
C10	0.7610 (4)	−0.0422 (2)	−0.06912 (13)	0.0236 (7)	
H10A	0.7078	−0.0974	−0.1017	0.028*	
C11	0.7849 (4)	−0.0914 (2)	0.00861 (14)	0.0223 (6)	
H11A	0.8562	−0.0415	0.0390	0.027*	
C12	0.8745 (4)	−0.2006 (2)	0.00551 (15)	0.0291 (7)	
H12A	0.7945	−0.2538	−0.0159	0.035*	
H12B	0.9764	−0.1956	−0.0270	0.035*	
H2	0.986 (6)	0.068 (4)	0.156 (2)	0.077 (15)*	
H3	0.881 (6)	0.333 (3)	0.184 (2)	0.050 (13)*	
H4	0.479 (5)	0.393 (3)	0.2228 (18)	0.041 (11)*	
H5	0.396 (5)	0.099 (3)	0.3545 (19)	0.053 (12)*	
H8	0.431 (6)	0.183 (3)	−0.007 (2)	0.064 (14)*	
H9	0.685 (5)	0.113 (3)	−0.1589 (19)	0.037 (11)*	
H10	0.959 (7)	0.037 (4)	−0.078 (2)	0.075 (18)*	
H11	0.852 (5)	−0.267 (3)	0.0975 (17)	0.039 (11)*	
H12C	0.987 (6)	0.151 (3)	0.046 (2)	0.068 (13)*	
H12D	1.114 (6)	0.156 (4)	−0.001 (2)	0.057 (15)*	
H13A	1.137 (7)	−0.094 (4)	0.191 (3)	0.085 (18)*	
H13B	1.045 (7)	−0.125 (4)	0.130 (3)	0.090 (17)*	

### Atomic displacement parameters (Å^2^)

**Table:**

	*U*^11^	*U*^22^	*U*^33^	*U*^12^	*U*^13^	*U*^23^
O1	0.0257 (11)	0.0192 (9)	0.0235 (9)	0.0023 (9)	0.0062 (9)	−0.0001 (8)
O2	0.0236 (12)	0.0371 (13)	0.0333 (11)	0.0024 (11)	0.0050 (9)	0.0025 (10)
O3	0.0360 (13)	0.0339 (13)	0.0353 (11)	−0.0152 (12)	0.0101 (11)	−0.0112 (11)
O4	0.0318 (13)	0.0201 (11)	0.0531 (13)	0.0038 (11)	−0.0005 (11)	−0.0016 (11)
O5	0.0345 (13)	0.0438 (13)	0.0214 (9)	0.0166 (12)	0.0034 (9)	−0.0013 (10)
O6	0.0283 (11)	0.0208 (9)	0.0185 (8)	−0.0012 (9)	0.0009 (8)	−0.0017 (8)
O7	0.0253 (11)	0.0182 (9)	0.0277 (9)	−0.0007 (9)	0.0066 (8)	−0.0012 (8)
O8	0.0194 (11)	0.0305 (12)	0.0471 (13)	0.0028 (10)	−0.0023 (10)	−0.0001 (10)
O9	0.0301 (14)	0.0540 (15)	0.0272 (12)	0.0088 (13)	0.0008 (10)	0.0146 (10)
O10	0.0305 (13)	0.0363 (13)	0.0344 (12)	0.0063 (12)	0.0136 (10)	0.0067 (11)
O11	0.0327 (13)	0.0334 (12)	0.0315 (12)	0.0010 (11)	0.0023 (10)	0.0125 (10)
O12	0.0253 (14)	0.0403 (13)	0.0411 (14)	−0.0014 (13)	0.0041 (11)	0.0050 (10)
O13	0.0624 (19)	0.0409 (14)	0.0477 (15)	0.0051 (15)	−0.0195 (14)	0.0037 (13)
C1	0.0278 (17)	0.0202 (14)	0.0201 (13)	0.0063 (13)	0.0058 (12)	0.0011 (11)
C2	0.0218 (16)	0.0303 (16)	0.0205 (12)	−0.0001 (14)	−0.0005 (12)	0.0012 (12)
C3	0.0276 (16)	0.0224 (14)	0.0204 (12)	−0.0058 (14)	0.0000 (12)	−0.0018 (12)
C4	0.0255 (16)	0.0222 (14)	0.0217 (13)	0.0015 (14)	0.0009 (12)	−0.0020 (11)
C5	0.0222 (15)	0.0218 (14)	0.0192 (12)	0.0016 (13)	−0.0010 (12)	−0.0040 (12)
C6	0.0247 (16)	0.0309 (16)	0.0264 (13)	0.0013 (14)	0.0003 (12)	−0.0041 (13)
C7	0.0190 (15)	0.0216 (13)	0.0283 (14)	−0.0043 (14)	0.0063 (12)	−0.0018 (11)
C8	0.0197 (15)	0.0245 (14)	0.0286 (14)	0.0018 (13)	−0.0010 (13)	−0.0037 (12)
C9	0.0243 (16)	0.0255 (14)	0.0195 (13)	−0.0001 (14)	−0.0014 (12)	0.0004 (12)
C10	0.0242 (17)	0.0258 (15)	0.0207 (13)	−0.0007 (13)	0.0014 (12)	−0.0009 (12)
C11	0.0217 (14)	0.0229 (14)	0.0223 (13)	−0.0013 (13)	0.0037 (12)	−0.0020 (11)
C12	0.0327 (18)	0.0267 (15)	0.0281 (14)	0.0028 (14)	0.0039 (14)	0.0025 (12)

### Geometric parameters (Å, º)

**Table:**

O1—C1	1.420 (3)	O13—H13B	0.88 (5)
O1—C5	1.438 (3)	C1—C2	1.514 (4)
O2—C2	1.426 (3)	C1—H1	0.9800
O2—H2	0.83 (4)	C2—C3	1.515 (4)
O3—C3	1.422 (4)	C2—H2A	0.9800
O3—H3	0.76 (4)	C3—C4	1.520 (4)
O4—C4	1.423 (3)	C3—H3A	0.9800
O4—H4	0.77 (4)	C4—C5	1.520 (4)
O5—C6	1.421 (3)	C4—H4A	0.9800
O5—H5	0.87 (4)	C5—C6	1.512 (4)
O6—C1	1.416 (3)	C5—H5A	0.9800
O6—C7	1.424 (3)	C6—H6A	0.9700
O7—C7	1.403 (3)	C6—H6B	0.9700
O7—C11	1.436 (3)	C7—C8	1.530 (4)
O8—C8	1.419 (3)	C7—H7	0.9800
O8—H8	0.83 (4)	C8—C9	1.517 (4)
O9—C9	1.427 (3)	C8—H8A	0.9800
O9—H9	0.75 (4)	C9—C10	1.511 (4)
O10—C10	1.439 (4)	C9—H9A	0.9800
O10—H10	0.76 (4)	C10—C11	1.526 (4)
O11—C12	1.421 (3)	C10—H10A	0.9800
O11—H11	0.78 (3)	C11—C12	1.501 (4)
O12—H12C	0.90 (4)	C11—H11A	0.9800
O12—H12D	0.72 (4)	C12—H12A	0.9700
O13—H13A	0.78 (5)	C12—H12B	0.9700
			
C1—O1—C5	113.98 (19)	O5—C6—C5	113.5 (2)
C2—O2—H2	109 (3)	O5—C6—H6A	108.9
C3—O3—H3	106 (3)	C5—C6—H6A	108.9
C4—O4—H4	111 (3)	O5—C6—H6B	108.9
C6—O5—H5	110 (2)	C5—C6—H6B	108.9
C1—O6—C7	115.8 (2)	H6A—C6—H6B	107.7
C7—O7—C11	114.3 (2)	O7—C7—O6	111.8 (2)
C8—O8—H8	111 (3)	O7—C7—C8	111.4 (2)
C9—O9—H9	107 (3)	O6—C7—C8	105.8 (2)
C10—O10—H10	106 (4)	O7—C7—H7	109.2
C12—O11—H11	111 (2)	O6—C7—H7	109.2
H12C—O12—H12D	104 (4)	C8—C7—H7	109.2
H13A—O13—H13B	109 (4)	O8—C8—C9	111.6 (2)
O6—C1—O1	112.1 (2)	O8—C8—C7	111.1 (2)
O6—C1—C2	106.3 (2)	C9—C8—C7	109.8 (2)
O1—C1—C2	110.0 (2)	O8—C8—H8A	108.1
O6—C1—H1	109.5	C9—C8—H8A	108.1
O1—C1—H1	109.5	C7—C8—H8A	108.1
C2—C1—H1	109.5	O9—C9—C10	110.9 (2)
O2—C2—C1	111.6 (2)	O9—C9—C8	109.1 (2)
O2—C2—C3	110.0 (2)	C10—C9—C8	111.6 (2)
C1—C2—C3	108.9 (2)	O9—C9—H9A	108.4
O2—C2—H2A	108.8	C10—C9—H9A	108.4
C1—C2—H2A	108.8	C8—C9—H9A	108.4
C3—C2—H2A	108.8	O10—C10—C9	109.8 (2)
O3—C3—C2	113.0 (2)	O10—C10—C11	109.3 (2)
O3—C3—C4	106.7 (2)	C9—C10—C11	112.0 (2)
C2—C3—C4	109.0 (2)	O10—C10—H10A	108.5
O3—C3—H3A	109.3	C9—C10—H10A	108.5
C2—C3—H3A	109.3	C11—C10—H10A	108.5
C4—C3—H3A	109.3	O7—C11—C12	106.8 (2)
O4—C4—C5	105.4 (2)	O7—C11—C10	111.3 (2)
O4—C4—C3	111.9 (2)	C12—C11—C10	111.8 (2)
C5—C4—C3	113.3 (2)	O7—C11—H11A	108.9
O4—C4—H4A	108.7	C12—C11—H11A	108.9
C5—C4—H4A	108.7	C10—C11—H11A	108.9
C3—C4—H4A	108.7	O11—C12—C11	112.4 (2)
O1—C5—C6	106.7 (2)	O11—C12—H12A	109.1
O1—C5—C4	111.6 (2)	C11—C12—H12A	109.1
C6—C5—C4	112.8 (2)	O11—C12—H12B	109.1
O1—C5—H5A	108.5	C11—C12—H12B	109.1
C6—C5—H5A	108.5	H12A—C12—H12B	107.9
C4—C5—H5A	108.5		

### Hydrogen-bond geometry (Å, º)

**Table:**

*D*—H···*A*	*D*—H	H···*A*	*D*···*A*	*D*—H···*A*
O2—H2···O13	0.83 (5)	1.91 (5)	2.727 (3)	168 (4)
O3—H3···O9^i^	0.76 (4)	2.05 (4)	2.754 (3)	153 (4)
O4—H4···O1^ii^	0.77 (4)	2.21 (4)	2.889 (3)	147 (3)
O5—H5···O10^iii^	0.87 (4)	1.88 (4)	2.726 (3)	163 (3)
O8—H8···O12^iv^	0.82 (4)	1.98 (4)	2.765 (3)	161 (4)
O9—H9···O4^i^	0.75 (4)	2.16 (4)	2.907 (3)	174 (4)
O10—H10···O12	0.75 (5)	2.14 (4)	2.887 (3)	170 (5)
O11—H11···O5^v^	0.78 (4)	1.94 (4)	2.709 (3)	173 (4)
O12—H12*C*···O2	0.89 (4)	1.87 (4)	2.766 (3)	175 (3)
O12—H12*D*···O8^vi^	0.72 (5)	1.99 (5)	2.709 (4)	171 (4)
O13—H13*A*···O3^vii^	0.78 (5)	2.04 (5)	2.801 (4)	169 (6)
O13—H13*B*···O11	0.88 (5)	1.89 (5)	2.765 (3)	179 (6)

Symmetry codes: (i) *x*+1/2, −*y*+1/2, −*z*; (ii) −*x*+1, *y*+1/2, −*z*+1/2; (iii) −*x*+3/2, −*y*, *z*+1/2; (iv) *x*−1/2, −*y*+1/2, −*z*; (v) −*x*+1, *y*−1/2, −*z*+1/2; (vi) *x*+1, *y*, *z*; (vii) −*x*+2, *y*−1/2, −*z*+1/2.
